# Identification of a six‐miRNA panel in serum benefiting pancreatic cancer diagnosis

**DOI:** 10.1002/cam4.2145

**Published:** 2019-04-21

**Authors:** Xuan Zou, Jishu Wei, Zebo Huang, Xin Zhou, Zipeng Lu, Wei Zhu, Yi Miao

**Affiliations:** ^1^ First Clinical College of Nanjing Medical University Nanjing PR China; ^2^ Pancreas Center First Affiliated Hospital of Nanjing Medical University Nanjing PR China; ^3^ Pancreas Institute, Nanjing Medical University Nanjing PR China; ^4^ Department of Oncology The Affiliated Hospital of Jiangnan University Wuxi PR China; ^5^ Department of Oncology The Affiliated Hospital of Nanjing Medical University Nanjing PR China; ^6^ Department of Oncology The Affiliated Jiangsu Shengze Hospital of Nanjing Medical University Suzhou PR China

**Keywords:** biomarker, pancreatic cancer, qRT‐PCR, serum miRNA

## Abstract

Pancreatic cancer (PC) has posed a great health threat to a growing number of people all over the world. Detection of serum miRNAs, being sensitive, noninvasive, and easy to obtain, has a great potential of being a novel screening method for PC patients. In this study, we investigated miRNA expression levels in serum by qRT‐PCR. The study was divided into four phases: the screening, training, testing, and external validation stage. We firstly chose candidate miRNAs using Exiqon panels in the screening phase. Then, a total of 129 PC serum samples and 107 normal controls (NCs) were further analyzed in the following training and testing phases to identify differently expressed miRNAs. A cohort of 30 PC serum samples vs 30 NCs was used to confirm the diagnostic value of the identified miRNAs in the external validation phase. Moreover, miRNA expressions in additional 44 PC tumor tissue samples and the matched adjacent normal tissue samples as well as 32 pairs of serum‐derived exosomes samples were also further explored. As a result, we identified six significantly upregulated miRNAs in the serum of PC: let‐7b‐5p, miR‐192‐5p, miR‐19a‐3p, miR‐19b‐3p, miR‐223‐3p, and miR‐25‐3p. A six‐miRNA panel in serum was then established. The area under the receiver operating characteristic curves (AUC) for the panel was 0.910 for the combined training and testing phases, which showed higher diagnostic value than the individual miRNA. Prognostic value prediction using Cox's proportional hazards model and Kaplan‐Meier curves showed that increased serum miR‐19a‐3p was closely related to worse overall survival (OS). In addition, significant upregulation of miR‐192‐5p, miR‐19a‐3p, and miR‐19b‐3p was observed in both PC tissue and serum‐derived exosomes samples. In conclusion, we identified a six‐miRNA (let‐7b‐5p, miR‐192‐5p, miR‐19a‐3p, miR‐19b‐3p, miR‐223‐3p, and miR‐25‐3p) panel in the serum for PC early and noninvasive diagnosis.

## 1 INTRODUCTION

Pancreatic cancer (PC) is a highly deadly disease with poor prognosis, for which mortality is almost equal to morbidity.^1^ Unlike most cancers with increasing survival rate, the 5‐year relative survival for PC currently remains as low as 8% in the USA.^2^ In China, the incidence rate of PC also presents a growing trend and has become a heavy health burden for Chinese patients.^3^ Surgical resection is considered to be the only option for cure, but most patients are asymptomatic until being diagnosed in the late stage and might miss the best chance of clinical operation.^4^ Conventional diagnostic methods like imaging technology and tumor biopsy still have some limitations such as relative more damage, higher price, and lower sensitivity or specificity.^5^ Thus, effective and noninvasive screening methods are in great demand to improve the overall survival for PC. Recently, biomarker‐based strategies have established their roles in the early detection of PC.^6^ Serum carbohydrate antigen 19‐9 (CA19‐9) is the only Food and Drug Administration (FDA)‐approved biomarker for PC diagnosis, but its nonspecific expression in other diseases, false‐negative results in Lewis negative phenotype and false‐positive elevation in the presence of obstructive jaundice have greatly limited its clinical application in PC management.^7^, ^8^ Therefore, it is quite necessary to discover novel tumor biomarkers which may provide more powerful and reliable diagnostic information for PC management.

MicroRNAs (miRNAs) are small noncoding RNAs with the length of about 18‐25 nucleotides and function in posttranscriptional regulation of gene expression and RNA silencing.^9^, ^10^ Aberrant miRNA expression continues to be observed in various diseases including cancers, and the roles miRNAs play in different disease processes have also been increasingly revealed.^11^ Moreover, the stable existence of many miRNAs has been discovered in various body fluids including serum and plasma, which makes it possible to monitor diseases through circulating miRNA analysis.^12^, ^13^ In recent years, a growing number of studies have concentrated on circulating miRNAs for their potential as noninvasive biomarkers for cancer diagnosis.^14^ As to PC, several circulating miRNAs were reported to be the possible biomarkers by different researchers.^15^, ^16^, ^17^, ^18^ However, the reported miRNAs were not always of high diagnostic value, and there was always little overlap between the results due to diverse methodologies or standards across laboratories.^19^ Therefore, more studies were required to identify promising miRNA signatures in blood circulation for PC diagnosis.

In this study, we analyzed serum miRNA expression patterns with Exiqon miRNA qPCR panel followed by three‐phase validation on the basis of quantitative real‐time polymerase chain reaction (qRT‐PCR). Meanwhile, miRNA expression levels in tissues and serum exosomes samples were also detected to explore the diagnostic potential of identified miRNAs. Hopefully, novel serum miRNA biomarkers with high specificity and sensitivity could be discovered for early diagnosis of PC.

## 2 METHODS AND MATERIALS

### 2.1 Patients and samples

A total of 159 histopathologically confirmed PC patients and 137 healthy donors who took routine health checkup were enrolled in this study. All the participants were recruited from First Affiliated Hospital of Nanjing Medical University during 2010 and 2014. Clinical characteristics of patients and healthy donors including gender, age, tobacco and alcohol addiction, disease history, CA19‐9 level, the location, and differentiation degree of tumor were recorded in detail. Tumor stages were determined according to the International Union Against Cancer's (UIAC) tumor‐node‐metastasis (TNM) system. The study was conducted with the approval of Institutional Review Boards of the First Affiliated Hospital of Nanjing Medical University. All the participants were previously untreated and had signed written consent forms.

Peripheral venous blood samples (5 mL) were collected in SST Advance tubes (Becton, Dickinson and Company). Then a two‐step centrifugal management (350 RCF [reactive centrifugal force] for 10 minutes and 20 000 RCF for 10 minutes [Beckman Coulter, USA]) were conducted to isolate serum from whole blood within 12 hours. Serum samples collected were restored at −80°C until use. Tumor and matched normal adjacent tissue specimens were obtained from PC patients undergoing surgical operation without preoperative radiochemotherapy and kept in liquid nitrogen.

### 2.2 Study design

A four‐phase procedure including the screening, training, testing, and the external validation phase was followed to identify potential miRNA biomarkers for PC diagnosis using qRT‐PCR. In the initial screening phase, miRNA expression patterns were performed using Exiqon miRCURY‐Ready‐to‐Use PCR‐Human‐panel‐I + II‐V1.M (Exiqon miRNA qPCR panel, Vedbaek, Denmark) which could detect 168 miRNAs in serum/plasma samples. In this stage, we randomly chose 20 serum samples from PC patients and 10 serum samples from normal controls (NCs) and formed two PC pools and one HC pool with per 10 samples grouped into one pool. miRNA profiles were performed following the procedure of previous study.^20^ Candidate miRNAs identified in the screening phase were then further analyzed in the training and testing phase using 36 PC serum samples vs 36 NCs and 93 PC serum samples vs 71 NCs, respectively. An external cohort of 30 PC patients and 30 NCs was finally used to confirm the diagnostic value of identified miRNAs. Moreover, miRNA expression levels were additionally analyzed among 44 pairs of tumor tissue samples and the matched adjacent normal tissue samples as well as 32 pairs of serum‐derived exosomes samples (32 PC vs 32 NCs).

### 2.3 Isolation of exosomes

ExoQuick Exosome Precipitation Solution (System Biosciences, Mountain View, CA) was used to extract exosomes from serum samples according to the manufacturer's protocol. Two hundred microliters of serum was mixed with 50 µL exosome precipitation solution and precipitated at 4°C overnight. Exosomes pellets were then obtained after centrifugation process of 1,500 g for 30 minutes and resuspended in 200 µL RNase‐free water ready for future analysis.

### 2.4 Extraction of total RNA

The mirVana PARIS Kit (Ambion, Austin, TX) was used to extract total RNA from 200 µL serum or exosomes following the given protocol. For normalization, 5 µL of synthetic *C. elegans* *miR‐39* (5 nM/L, RiboBio, Guangzhou, China) was added to each sample after the addition of denaturing solution (Ambion, Austin, TX). Tissue samples were processed using Trizol (Invitrogen, Carlsbad, CA) to extract total RNA. Acquired total RNA was lysed in 100 µL RNase‐free water and restored at −80°C until use. The NanoDrop ND‐1000 spectrophotometer (NanoDrop, Wilmington, DE) was used to measure the concentration and purity of RNA.

### 2.5 Quantitative real‐time polymerase chain reaction

miRNA amplification was conducted using Bulge‐Loop™ miRNA qRT‐PCR Primer Set (RiboBio, Guangzhou, China) according to the manufacturer's protocol. In brief, miRNA was firstly reverse transcribed (RT) to complementary DNA (cDNA) in the condition of 42°C for 60 minutes followed by 70°C for 10 minutes. The following polymerase chain reactions (PCR) were carried out in triplicate in 384‐well plates on at 95°C for 20 seconds, followed by 40 cycles of 95°C for 10 seconds, 60°C for 20 seconds, and then 70°C for 10 seconds. A 7900HT real‐time PCR system (Applied Biosystems, Foster City, CA) was applied to amplify miRNAs and SYBR Green dye (SYBR® Premix Ex Taq^TM^ II, TaKaRa, Dalian, China) was used to evaluate the amount of PCR products. Standard melting curve was constructed using the synthetic miRNAs (micrON miRNA mimic, RiboBio, Guangzhou, China). The relative expression levels of miRNAs to reference miRNA (serum and exosomes: *cel‐miR‐39*; tissue: *RNU6B (U6)*) were calculated using the 2^−ΔΔCt^ method.^21^, ^22^

### 2.6 Analysis of statistics

The Mann‐Whitney test was used to compare the expression levels of serum miRNAs between PC patients and NCs. One‐way ANOVA or chi‐squared test was applied to detect demographic and clinical characteristics among groups and their relationship with miRNA expression levels. Multiple comparison among separate independent phases was conducted using Kruskal‐Wallis rank test. Multiple logistic regression analysis was conducted for the establishment of miRNA signature. The diagnostic value of the identified serum miRNAs and the signature was evaluated by receiver operating characteristic (ROC) curves and the area under the ROC curve (AUC). The corresponding prognostic value was estimated by overall survival (OS) rate. Factors related to the OS were assessed using Cox's proportional hazards model and the association between identified miRNAs and OS was estimated by Kaplan‐Meier curves using logrank test. The software SPSS version 22.0 for windows (SPSS Inc, Chicago, IL) and GraphPad Prism 7.0 (GraphPad Software, USA) were applied for data analysis and graph drawing. A two‐sided *P* < 0.05 was considered to be of statistical significance.

## 3 RESULTS

### 3.1 Characteristics of the study cohort

**Figure 1:**
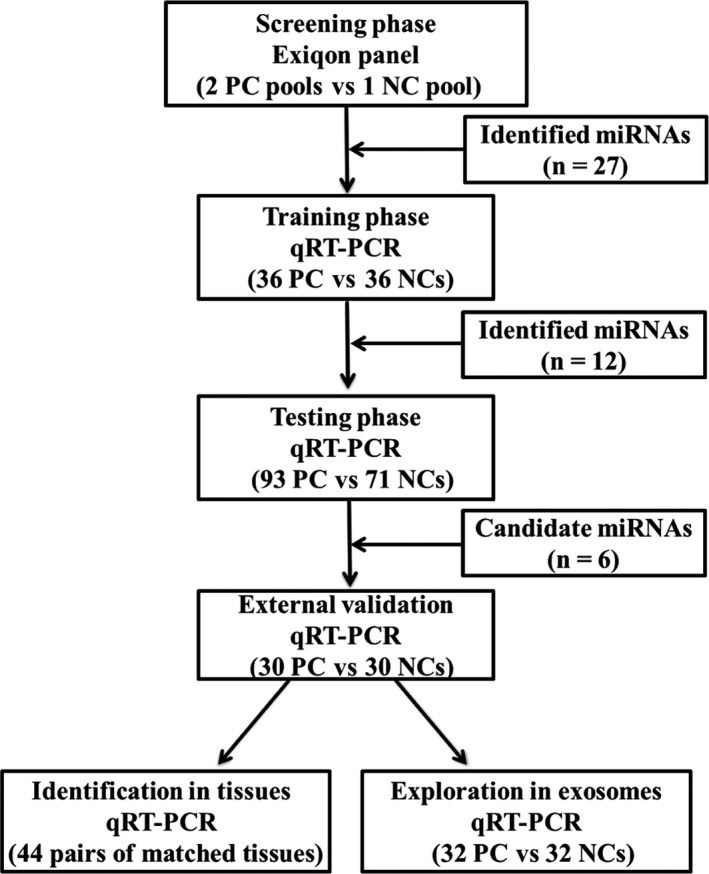
The flow chart of experiment design. PC: pancreatic cancer; NC: normal control

The study was designed into four phases to explore potential miRNA biomarkers in serum for PC diagnosis (Figure 1). The objects of the study included 157 PC patients and 137 NCs in all, whose characteristics in three independent phases were given in Table 1. In order to eliminate selection bias, several potential factors such as the ratio of sample sizes between case and control groups, gender, age, tobacco and alcohol addiction, diabetes mellitus as well as different disease states were taken into full consideration and evenly distributed across four phases as possible. The distribution of gender and age has no significant difference between PC cases and NCs, while different clinical features composition was relatively even between different phases (*P* > 0.05).

**Table 1:** Characteristics of 159 PC patients and 137 NCs enrolled in the study

Variables	Training and testing phases (n = 80)		External validation cohort (n = 60)	
	Cases (%)	Controls (%)	Cases (%)	Controls (%)
Number	129	107	30	30
Gender				
Male	80 (62.0)	69 (64.5)	18 (60.0)	15 (50.0)
Female	49 (38.0)	38 (35.5)	12 (40.0)	15 (50.0)
Age				
≤60	33 (25.6)	46 (43.0)	11 (36.7)	13 (43.3)
>60	96 (74.4)	61 (57.0)	19 (63.3)	17 (56.7)
Smoking				
Smoker	32 (24.8)		12 (40.0)	
Nonsmoker	87 (67.4)		15 (50.0)	
NA	10 (7.8)		3 (10.0)	
Drinking				
Drinker	23 (17.8)		7 (23.3)	
Nondrinker	95 (73.7)		16 (53.3)	
NA	11 (8.5)		7 (23.3)	
Diabetes mellitus				
Yes	27 (20.9)		8 (26.7)	
No	95 (73.7)		18 (60.0)	
NA	7 (5.4)		4 (13.3)	
Differential				
Well	51 (39.5)		9 (30.0)	
Poor	56 (43.4)		16 (53.3)	
NA	22 (17.1)		5 (16.7)	
Location				
Head	58 (45.0)		13 (43.3)	
Body or tail	56 (43.4)		9 (30.0)	
NA	15 (11.6)		8 (26.7)	
CA199				
Elevation	59 (45.7)		16 (53.3)	
Normal	25 (19.4)		9 (30.0)	
NA	45 (34.9)		5 (16.7)	
TNM stage				
I	13 (10.1)		3 (10.0)	
II	65 (50.3)		13 (43.3)	
III	6 (4.7)		5 (6.7)	
IV	29 (22.5)		8 (26.7)	
NA	16 (12.4)		1 (3.3)	

PC: pancreatic cancer; NA: not available

### 3.2 Discovery of candidate miRNAs in the screening phase

In the screening phase, miRNA profiles of the two serum pools from 20 PC cases and one serum pool from 10 NCs were analyzed based on the Exiqon miRCURY‐Ready‐to‐Use‐PCR‐Human‐panel‐I + II‐V1.M. In this panel, miRNAs with the cycle threshold (Ct) value < 37 and 5 lower than negative control (No Template Control, NTC) were included in data analysis. Among the 168 relatively abundant miRNAs in serum/plasma, we identified 27 differently expressed serum miRNAs of which 25 miRNAs were upregulated (more than 1.5‐fold) and two miRNAs were downregulated (less than 0.67‐fold) in both two PC pool samples compared with NCs (Supplementary Table S1). These miRNAs were selected for further analysis in the following phases.

### 3.3 Confirmation of candidate miRNAs by qRT‐PCR

**Figure 2:**
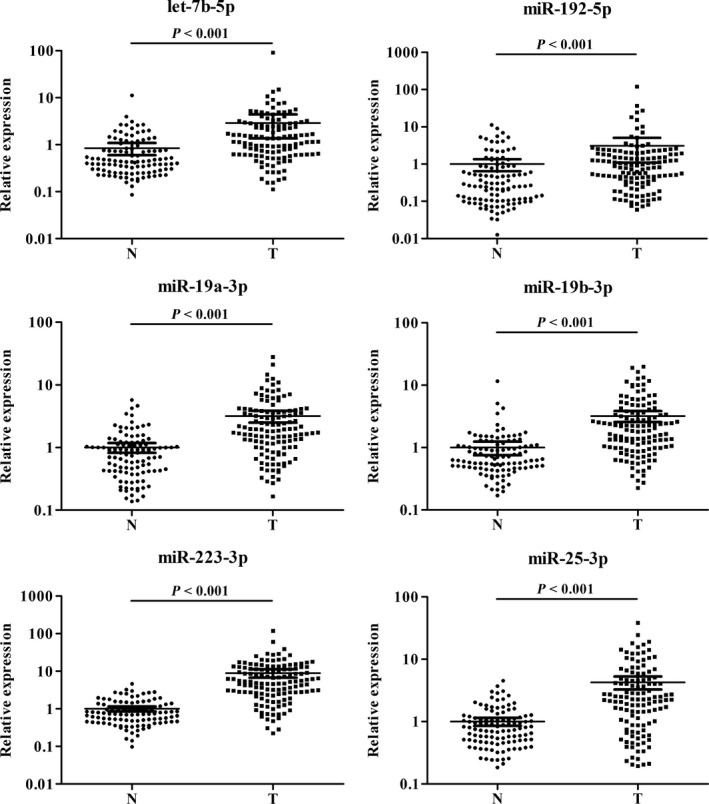
Expression levels of six miRNAs in the serum of 129 PC patients and 107 NCs (in the training and testing phases). N: normal control; T: tumor. Horizontal line: mean with 95% CI

In the following training phase, we first analyzed the miRNA expression levels in 36 PC serum samples vs 36 NCs by qRT‐PCR. Among the 27 candidate miRNAs, twelve miRNAs (let‐7b‐5p, miR‐122‐5p, miR‐151a‐3p, miR‐192‐5p, miR‐19a‐3p, miR‐19b‐3p, miR‐2110, miR‐223‐3p, miR‐25‐3p, miR‐483‐5p, miR‐486‐5p and miR‐877‐5p) were still significantly dysregulated in the serum of PC patients compared with NCs (fold change (FC) >1.5 or <0.67). These miRNAs were further detected in the testing phase using 93 PC serum samples vs 71 NCs by qRT‐PCR. In the larger cohort, six miRNAs (let‐7b‐5p, miR‐192‐5p, miR‐19a‐3p, miR‐19b‐3p, miR‐223‐3p and miR‐25‐3p) of the 12 miRNAs were still upregulated (FC > 2) in the case group in comparison with NCs. As is shown in Figure 2, when the training and testing phases were combined, the six miRNAs showed the same expression feature of upregulation as in the two separated phases (*P* > 0.05). We further conducted multiple comparison among training, testing, and the following external validation phases (as hereunder mentioned), which statistically verified the consistency and stability of expression levels of the six identified miRNAs for both PC patients and NCs among different phases with *P* > 0.05.

To avoid potential confounding effect of some other clinical features such as gender, age, smoking, drinking, and diabetes mellitus, we further performed subgroup analysis among PC patients. Actually, none of these factors had significant influence on serum miRNA expression for PC patients (*P > *0.05; data not shown), which further demonstrated the dependability of these identified miRNA signatures.

### 3.4 Diagnostic value of identified miRNAs in serum

We performed ROC analysis and calculated AUC values for each of the six identified miRNAs to evaluate their diagnostic values for PC. In combination of the training and testing phases, the AUCs for let‐7b‐5p, miR‐192‐5p, miR‐19a‐3p, miR‐19b‐3p, miR‐223‐3p and miR‐25‐3p were 0.703 (95% CI: 0.636‐0.771; sensitivity = 79.8%, specificity = 59.8%), 0.684 (95% CI: 0.615‐0.754; sensitivity = 77.5%, specificity = 57.0%), 0.771 (95% CI: 0.712‐0.831; sensitivity = 71.3%, specificity = 78.5%), 0.788 (95% CI: 0.729‐0.846; sensitivity = 65.1%, specificity = 81.3%), 0.901 (95% CI: 0.861‐0.941; sensitivity = 78.3%, specificity = 91.6%), and 0.726 (95% CI: 0.659‐0.792; sensitivity = 66.7%, specificity = 80.4%), respectively (Supplementary Figure S1).

**Figure 3:**
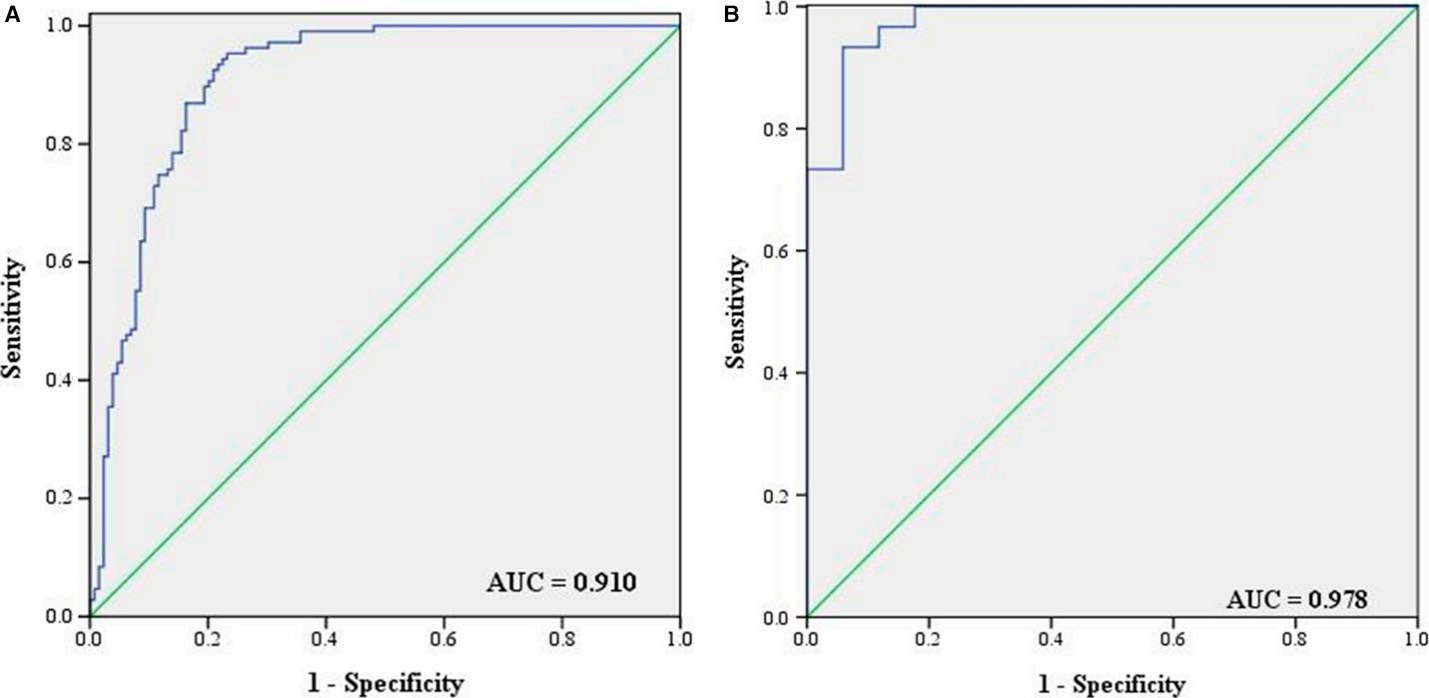
ROC curve analyses of the six‐miRNA signature to discriminate PC patients from NCs. (A) The combined two cohorts of training and testing phases (129 PC vs 107 NCs); (B) external validation phase (30 PC vs 30 NCs). AUC: areas under the curve; ROC curve: receiver operating characteristic curve

In addition, we combined the sixserum miRNAs together and formed a six‐miRNA panel to compare its diagnostic performance for PC with single miRNA. Predicted probability of PC detection by the panel in a logistic regression model was calculated with the formula: Logit(P) = 2.358 + 0.149 × let‐7b − 0.052 × 192 + 0.040 × 19a + 0.007 × 19b − 0.936 × 223 − 0.089 × 25. The corresponding ROC analysis for the panel was also conducted and the AUC was 0.910 (95% CI: 0.872‐0.948; sensitivity = 95.3%, specificity = 76.7%; Figure 3A) for the combined two cohorts of training and testing phases.

In the external validation phase, an additional cohort of 30 PC samples and 30 NCs were analyzed for further validation of the diagnostic capacity of the six‐miRNA signature. Similar to the previous training and testing phases, the six identified miRNAs showed consistent tendency of upregulation (Supplementary Figure S2) and the panel still performed quite well in discriminating PC patients from NCs with the AUC being 0.978 (95% CI: 0.966‐0.998; sensitivity = 93.3%, specificity = 96.0%; Figure 3B) in this phase.

### 3.5 Prognostic value of identified miRNAs for PC

For survival analysis, we constructed Cox proportional hazards model and Kaplan‐Meier curves to estimate the prognostic value of the six miRNAs and the association between clinical influence factors and OS. According to univariate Cox regression analysis and the following multivariate analysis, vascular/nerve infiltration, positive lymph node, and increased serum miR‐19a‐3p level were considered as independent predictive factors (*P* < 0.05) for worse OS of PC patients (Table 2), with the formula of Cox proportional hazards model being *h*(*t,X*) = *h*_0_(*t*) exp(0.600 × vascular/nerve infiltration + 0.507 × lymph node + 0.845 × tumor stage + 0.450 × miR‐19a‐3p). Moreover, among the six identified miRNAs in serum, only higher miR‐19a‐3p expression level in the serum was significantly correlated with worse OS according to the results of Kaplan‐Meier curve analysis (Supplementary Figure S3).

**Table 2:** The relationship between OS and clinical factors for PC

Variables	Univariate analysis		Multivariate analysis		
	HR (95% CI）	*P* value	HR (95% CI)	*P* value	
Gender (female vs male)	0.632 (0.316, 1.266)	0.196			
Age (>60 vs ≤60)	0.601 (0.306, 1.180)	0.139			
Location (head vs body/tail)	0.559 (0.239, 1.308)	0.180			
Size (≥35 vs <35)	0.693 (0.361, 1.332)	0.271			
**Vascular nerve infiltration (yes vs. no)**	**3.266 (1.442, 7.395)**	**0.005**	**1.822 (1.063, 3.122)**		**0.029**
Resection margin (yes vs no)	4.605 (0.652, 32.552)	0.126			
Smoking (yes vs no)	1.767 (0.544, 5.740)	0.344			
Drinking (yes vs no)	0.457 (0.147, 1.415)	0.174			
DM (yes vs no)	0.799 (0.318, 2.008)	0.633			
T (3 + 4 vs 1 + 2)	0.419 (0.168, 1.043)	0.062			
**N (yes vs no)**	**3.138 (1.362, 7.229)**	**0.007**	**1.661 (1.041, 2.649)**		**0.033**
**TNM stage (III + IV**vs** I + II)**	**5.797 (2.612, 12.867)**	**0.000**	**2.327 (1.405, 3.856)**		**0.001**
CA199 (≥median vs <median)	1.491 (0.714, 3.113)	0.287			
let‐7b‐5p (≥median vs <median)	1.487 (0.503, 4.398)	0.473			
miR‐192‐5p (≥median vs <median)	0.707 (0.309, 1.615)	0.410			
**miR‐19a‐3p (≥median vs <median)**	**3.125 (1.078, 9.091)**	**0.036**	**1.568 (1.149, 2.442)**		**0.042**
miR‐19b‐3p (≥median vs <median)	0.657 (0.270, 1.597)	0.354			
miR‐223‐3p (≥median vs <median)	1.523 (0.317, 7.311)	0.599			
miR‐25‐3p (≥median vs <median)	1.811 (0.535, 6.129)	0.340			
Predicted point (≥median vs <median)	2.375 (0.614, 9.186)	0.210			

Cox regression analysis identified four independent predictive factors associated with worse prognosis of PC. In this study, patients with higher serum miR‐19a‐3p levels had significantly worse OS compared to those with lower levels. However, no significant difference was found in other five identified miRNAs. Other clinical factors including positive vascular nerve infiltration status, lymph node metastasis and higher TNM stage (III or IV) were also proved to be associated with worse PC prognosis (in bold).

PC: pancreatic cancer; OS: overall survival; HR: hazard ratio; CI: confidence interval; DM: diabetes mellitus; T: tumor topography; N: lymph node.

### 3.6 miRNA expression in tissue specimens

**Figure 4:**
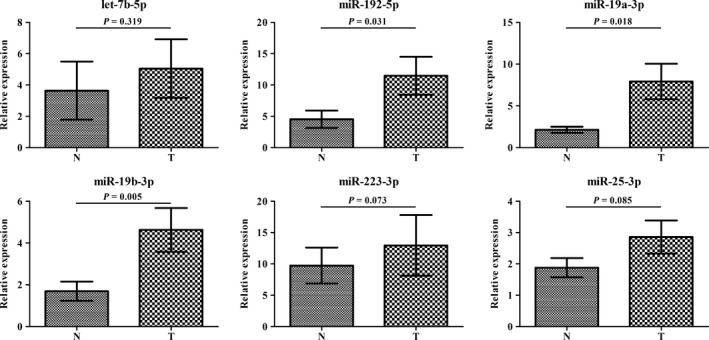
Expression levels of six miRNAs in 44 PC tumor tissues and the matched adjacent normal tissues. T: tumor; N: normal control

Expression levels of the six identified miRNAs were also detected in 44 pairs of tissues samples (44 PC tumor tissues and the matched adjacent normal tissues). As shown in Figure 4, miR‐192‐5p, miR‐19a‐3p, and miR‐19b‐3p were significantly upregulated in tumor tissues than in normal tissues (*P* < 0.05). No significant difference was observed in the other three miRNAs.

### 3.7 miRNA expression in serum exosomes

**Figure 5:**
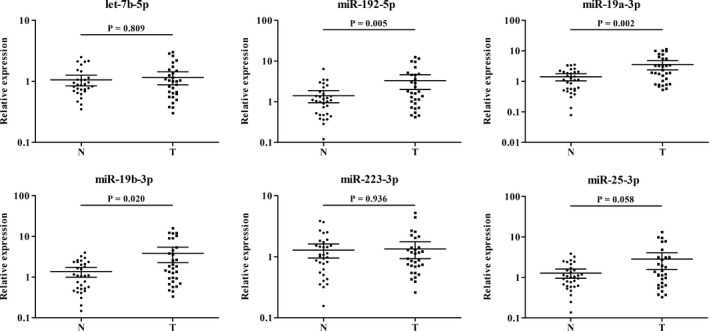
Expression levels of six miRNAs in 32 PC serum‐derived exosomes and 32 NCs. T: tumor; N: normal control

To explore the potential existence form of the identified miRNAs, we isolated exosomes from serum samples of 32 PC patients and 32 NCs and investigated miRNA expression levels by qRT‐PCR. Consistent with the results in tissue, significant higher levels of miR‐192‐5p, miR‐19a‐3p, and miR‐19b‐3p were observed in serum‐derived exosomes in PC patients than in NCs (Figure 5).

### 3.8 Bioinformatics analysis of the six identified miRNAs

**Figure 6:**
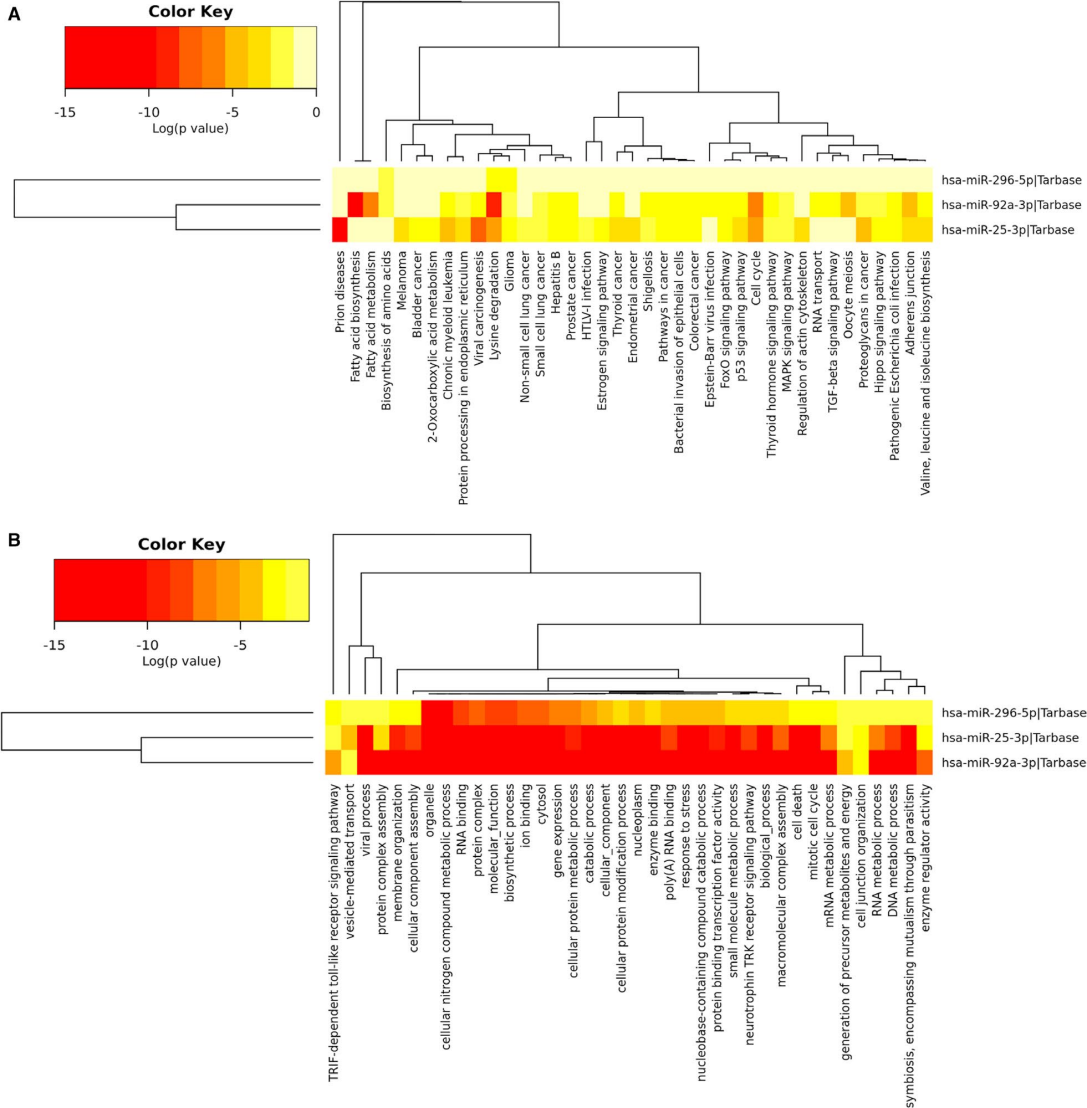
Heat‐maps of pathway investigation using KEGG (A) and GO (B) analyses. KEGG: Kyoto Encyclopedia of Genes and Genomes; GO: Gene Ontology

We applied an online miRNA pathway analysis web‐server DIANA‐miRPath v3.0 *(* http://www.microrna.gr/miRPathv3 *)* to decipher the functional characterization of the six miRNAs based on experimentally validated miRNA interactions derived from DIANA‐TarBase7.0.^23^ Heat‐maps of pathway investigation using Kyoto Encyclopedia of Genes and Genomes (KEGG) and Gene Ontology (GO) analyses are given in Figure 6. KEGG analysis revealed several cancer‐related pathways regulated by the six miRNAs such as proteoglycans in cancer, transcriptional dysregulation in cancer, cell cycle and p53 signaling pathway (Figure 6A). The results of GO analysis showed intersection of the pathways related to each identified miRNA. These miRNAs all had close relationship with processes including response to stress, cellular protein modification process, and Fc‐epsilon receptor signaling pathway, which might indicate their biological functions in common (Figure 6B).

## 4 DISCUSSION

During the last decades, PC has constituted a serious health risk to people worldwide, whose mortality rate nearly equals the incidence rate.^1^ To some extent, it is the discovery of new early detection methods rather than new therapeutic agents that is more helpful to improve overall prognosis for PC patients.^24^ Recently, an increasing number of novel biomarkers or molecular targets have constantly emerged for PC diagnosis, including circulating miRNAs. Stable existence in peripheral circulation makes it possible for plenty of miRNAs to serve as promising biomarkers for various diseases including cancers.^25^, ^26^ The role of circulating miRNAs in the diagnosis and management of PC have been revealed by several reports. For example, Schultz et al identified 38 dysregulated miRNAs (miR‐145, miR‐150, miR‐223, and so on) in whole blood and constructed two diagnostic panels to discriminate PC patients from healthy controls.^27^ Miyamae et al discovered plasma miR‐744 as a useful biomarker for screening PC.^28^ Liu et al demonstrated seven miRNAs (miR‐20a, miR‐21, miR‐24, and so on) in serum which could be a novel noninvasive approach for PC diagnosis. However, there was poor consistence between these results and the signatures identified were not always of high specificity and sensitivity. Moreover, few researches have been done to explore miRNA expression profiles especially for Chinese PC patients. Hopefully, our study might provide some valuable reference for clinical diagnostic and prognostic screening for PC.

We performed a four‐phase study to identify differently expressed miRNAs in serum. In the screening phase, we applied Exiqon miRNA qPCR panels which could detect 168 miRNAs in the serum to select candidate miRNAs for further analysis. Compared to the other commonly used TaqMan Human MicroRNA Array v3.0. platform, the miRCURY platform showed better sensitivity and linearity in the condition of lower concentration range.^29^ A total of 27 serum miRNAs (25 miRNAs upregulated and two miRNAs downregulated) were screened out into the next step. In the training and testing phases, we analyzed a total of 129 serum samples from PC patients and 107 from NCs. miRNA signatures were evaluated using qRT‐PCR. As a result, six miRNAs (let‐7b‐5p, miR‐192‐5p, miR‐19a‐3p, miR‐19b‐3p, miR‐223‐3p, and miR‐25‐3p) were confirmed to be consistently upregulated in the serum of PC patients. ROC curves were constructed and AUCs were calculated to evaluate the diagnostic accuracy of each identified miRNA. When the two phases were combined, the AUCs for let‐7b‐5p, miR‐192‐5p, miR‐19a‐3p, miR‐19b‐3p, miR‐223‐3p, and miR‐25‐3p were 0.703, 0.684, 0.771, 0.788, 0.901, and 0.726, respectively. Since many previous studies indicated that the combination of miRNA biomarkers might have better diagnostic performance than single one, we then constructed a six‐miRNA panel in the serum and conducted ROC analysis to compare its diagnostic accuracy with each identified miRNA.^30^, ^31^, ^32^ Surprisingly, the AUC for the panel was 0.910 when the training and testing phases were combined. The external validation phase further demonstrated the accuracy and reliability of the six‐miRNA signature in detecting PC.

In addition, prognostic value of identified miRNAs was also evaluated by performing Cox's proportional hazards model and Kaplan‐Meier curves. We found that the high level of miR‐19a‐3p in the serum could act as independent predictor of worse OS, besides other influencing factors including vascular/nerve infiltration and positive lymph node. miRNA expression patterns in tissue were further explored among 44 PC tumor and the matched normal tissue specimens. miR‐192‐5p, miR‐19a‐3p, and miR‐19b‐3p were consistently upregulated in tumor tissues as in serum, indicating their potential mechanisms in regard to tumor biological processes.

miRNAs are highly stable molecules which assume both intracellular and extracellular forms. Some miRNAs are highly enriched within cells, and the dysregulation of some specific miRNAs in tumor cells in comparison with normal cells may indicate their regulatory functions in cancer development.^33^ Some extracellular miRNAs are released from cells via different types of vesicles, which may indicate possible cell‐to‐cell communication.^34^, ^35^ Numerous studies have focused on the roles miRNAs play in various cancers. The aberrantly regulated miRNAs may function as tumor promotors or suppressors by affecting processes of cell proliferation, apoptosis, differentiation, and migration in different human cancers.^36^, ^37^ In our study, among the six identified miRNAs, miR‐192‐5p, miR‐19a‐3p, and miR‐19b‐3p were significantly upregulated in both PC serum and tumor tissue samples. Serum miR‐19a as biomarker for cancer diagnosis was once reported in breast cancer by Sochor et al.^38^ But its dysregulation in PC has rarely been reported. In this study, we not only observed significant upregulation of miR‐19a‐3p in both serum and tissue in PC patients, but also preliminary forecasted the relationship of higher miR‐19a‐3p level in serum with relatively worse prognosis for PC. The results captured the imagination of the role miR‐19a‐3p plays in disease process of PC. According to Tan et al, higher levels of miR‐19a were found in PC tissues and miR‐19a was confirmed to have tumor‐promoting effect of stimulating cell proliferation, migration, invasion in vitro and tumor growth in vivo by targeting RHOB in PC.^39^ Besides PC, miR‐19a is also frequently overexpressed in tumor cells and acts as tumor‐promoting factor in other cancer types such as lung carcinoma, colorectal cancer, cervical carcinoma, and gastric cancer.^40^, ^41^, ^42^, ^43^ All of these findings give us strong hint to the function of miR‐19a‐3p which is highly worth exploring.

miR‐19b‐3p is another member of the miR‐17‐92 cluster.^44^ It has been frequently recognized as onco‐miR and was found to be amplified in multiple tumor types.^45^ In Jiang's study, miR‐19b‐3p was found to be high expressed in colon cancer cells and could promote proliferation and chemoresistance by targeting SMAD4. According to Wu et al, miR‐19a/b was overexpressed in gastric cancer tissues and could promote metastasis by regulating tumor suppressor MXD1.^46^ However, according to another study, miR‐19‐3p showed to be a tumor suppressor in breast cancer by inhibiting cancer cell proliferation and changing saracatinib resistance status via targeting PI3K/Akt pathway.^47^ It indicates the duplicity of miR‐19b‐3p in cancers.^48^ Circulating miR‐19b‐3p has also been found to be reliable biomarker for various diseases. For example, higher level of plasma miR‐19b‐3p was discovered in gastric cancer and might serve as indicators of gastric cancer progression.^49^ But little study has focused on the expression pattern of circulating miR‐19b‐3p in PC or the role of miR‐19b‐3p in the biological processes of PC.

As to miR‐192‐5p, upregulation in blood circulation in liver injury‐related diseases has been reported by several studies.^50^, ^51^, ^52^, ^53^ In liver fluke‐associated cholangiocarcinoma, serum miR‐192‐5p level was also elevated and might be involved in tumor genesis, metastasis, and poor survival.^54^ For some cancers such as hepatocellular carcinoma, bladder cancer, and non‐small cell lung cancer, miR‐192‐5p could act as a promoter of tumor proliferation and metastasis by targeting semaphorin 3A, Yin Yang 1, and the PI3K/Akt pathway, respectively.^55^, ^56^, ^57^ Besides, the other three upregulated serum miRNAs (let‐7b‐5p, miR‐223‐3p, and miR‐25‐3p) have also been reported to be associated with various cancer types, including prostate cancer, ovarian cancer, lung cancer, multiple myeloma, and anaplastic thyroid carcinoma.^58^, ^59^, ^60^, ^61^, ^62^, ^63^ In fact, researches focusing on the roles these identified miRNAs played in PC were still insufficient and in great demand. Findings from previous studies could be a reference for future functional exploration. What's more, bioinformatics analysis could also provide some clues to the potential roles of miRNAs in PC development.

Exosomes are small membrane‐bound vesicles released by many cell types and exist in almost all body fluid types.^64^ Circulating miRNAs have been detected in exosomes in a stable form.^65^ Since exosomes play very important roles in cell‐to‐cell communication, exosomes‐carried miRNAs can not only become reliable biomarkers but also indicate underlying molecular processes for cancers. Therefore, to explore the possible forms of identified miRNAs, we also detected their expression levels in serum‐derived exosomes. Interestingly, miR‐192‐5p, miR‐19a‐3p, and miR‐19b‐3p were still upregulated in exosomes as in serum and tissues. Though exosome‐encapsulated miR‐223‐3p was once reported to be noninvasive biomarker for breast cancer, no significant statistical difference was observed for PC in this study.^66^ To date, there were still few reports being published on the exosomes miRNAs characteristics of PC patients. We suspected that the partial consistency across various sample types in our study could be due to the release of intracellular miRNAs from parental cells via microvesicles such as exosomes during the process of which the changes of PC disease states happened. The exact function waited more study.

In this study, we identified a six‐miRNA panel in serum for PC diagnosis. It may serve as reliable and noninvasive biomarkers for PC in the future. However, some limitations of this study could not be ignored as well. For example, no suitable endogenous reference was confirmed for between‐sample normalization, which might lead to inevitable systematic errors. Moreover, the function of these miRNAs and their relationship with different clinical characteristics in PC were still unclear and further study subsequently is still in need. In the end, there would still be a long way to go before future clinical application. Serum samples from 159 PC patients and 137 healthy controls were far from being adequate to testify the positive results. Validation among larger samples is absolutely necessary for the confirmation of the identified markers. In fact, we are now making efforts to enroll more PC patients and verify diagnostic value of the signature before clinical application in the future. The present study, with its impressive specificity and sensitivity of the identified markers, can be the first step to further exploration.

## 5 CONCLUSION

In conclusion, we identified a six‐miRNA signature in the serum for PC detection, which contained let‐7b‐5p, miR‐192‐5p, miR‐19a‐3p, miR‐19b‐3p, miR‐223‐3p, and miR‐25‐3p. We expected the panel to become a stable, reliable, and valuable diagnostic method for PC patients. Since the exact mechanisms were still unrevealed, more researches on the identified miRNAs in relation to PC were in need in the future.

## ETHICS APPROVAL AND CONSENT TO PARTICIPATE

All procedures were approved by Institutional Review Boards of the First Affiliated Hospital of Nanjing Medical University. Written informed consent was obtained from patients involved in the study.

## CONFLICT OF INTERESTS

The authors declare that they have no conflict of interest.
