# Supplementary material for: Identification of a six‐miRNA panel in serum benefiting pancreatic cancer diagnosis
Source: Cancer Med. 2019 Apr 21;8(6):2810–22. doi: 10.1002/cam4.2145 (PMC6558458; doi:10.1002/cam4.2145)

**Table S1. Differently expressed miRNAs in the screening phase**

| **miRNA** | **Fold change** | | |
| --- | --- | --- | --- |
|  | Pool 1 | Pool 2 | Mean fold |
| let-7b-5p | 8.23 | 2.96 | 5.60 |
| miR-122-5p | 11.90 | 17.46 | 14.68 |
| miR-146b-5p | 2.95 | 1.59 | 2.27 |
| miR-151a-3p | 10.57 | 4.36 | 7.47 |
| miR-152-3p | 7.17 | 4.76 | 5.96 |
| miR-192-5p | 13.46 | 12.59 | 13.03 |
| miR-194-5p | 8.32 | 4.38 | 6.35 |
| miR-195-5p | 7.51 | 4.82 | 6.17 |
| miR-19a-3p | 7.88 | 9.53 | 8.70 |
| miR-19b-3p | 5.92 | 3.49 | 4.71 |
| miR-2110 | 8.64 | 3.62 | 6.13 |
| miR-215-5p | 5.68 | 3.41 | 4.54 |
| miR-21-5p | 7.34 | 4.66 | 6.00 |
| miR-223-3p | 5.84 | 2.24 | 4.04 |
| miR-223-5p | 6.41 | 3.36 | 4.88 |
| miR-23a-3p | 7.06 | 4.02 | 5.54 |
| miR-25-3p | 9.46 | 5.24 | 7.35 |
| miR-30a-5p | 6.12 | 2.08 | 4.10 |
| miR-30d-5p | 5.47 | 2.52 | 3.99 |
| miR-339-5p | 3.63 | 2.65 | 3.14 |
| miR-483-5p | 3.51 | 5.34 | 4.43 |
| miR-486-5p | 6.88 | 3.42 | 5.15 |
| miR-574-3p | 16.25 | 10.83 | 13.54 |
| miR-7-1-3p | 7.75 | 3.85 | 5.80 |
| miR-877-5p | 4.41 | 3.35 | 3.88 |
| miR-106b-5p | -3.29 | -2.46 | -2.88 |
| miR-144-3p | -2.53 | -2.40 | -2.47 |

**Figure S1.** Receiver-operating characteristic (ROC) curve analyses of the six serum miRNAs to discriminate PC patients from NCs in the combined training and testing phases. AUC: areas under the curve.

**
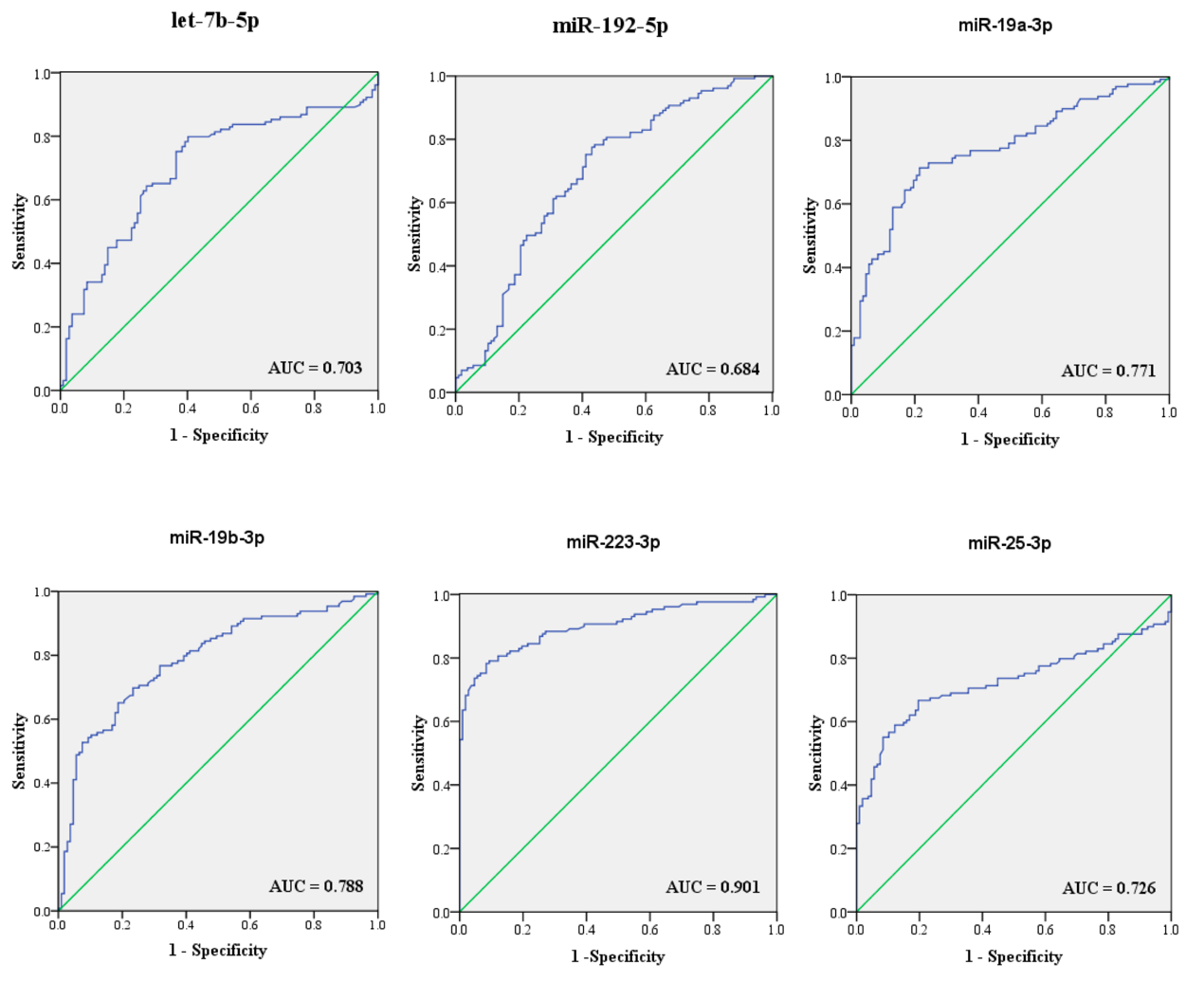
**

**Figure S2.** Expression levels of the six miRNAs in serum of 30 PC patients and 30 NCs in the external validation phase. PC: pancreatic cancer; NC: normal control; T: tumor. Horizontal line: mean with 95% CI.


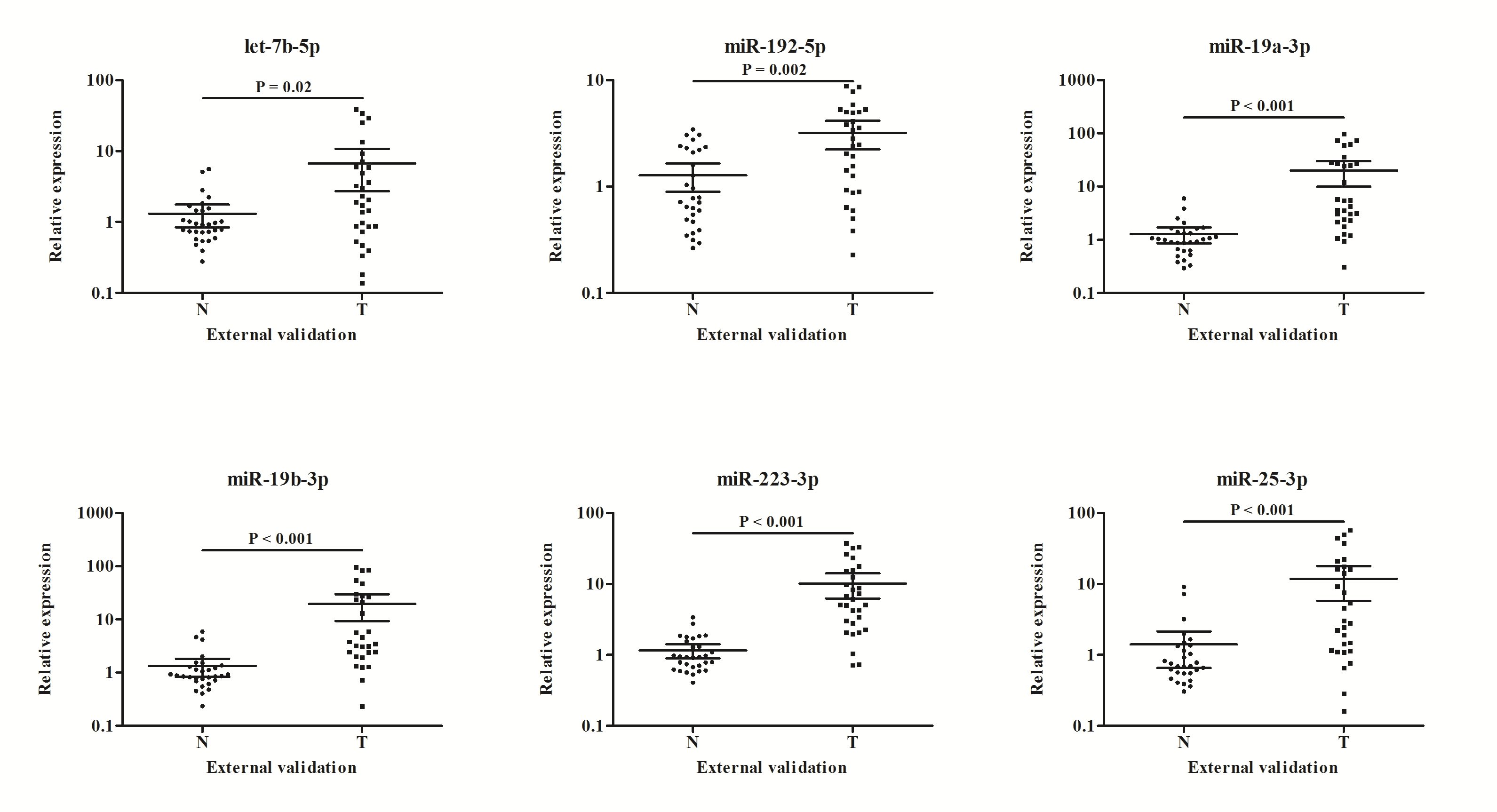


**Figure S3.** Kaplan–Meier curves estimating the association of the six miRNAs and the overall survival of PC patients


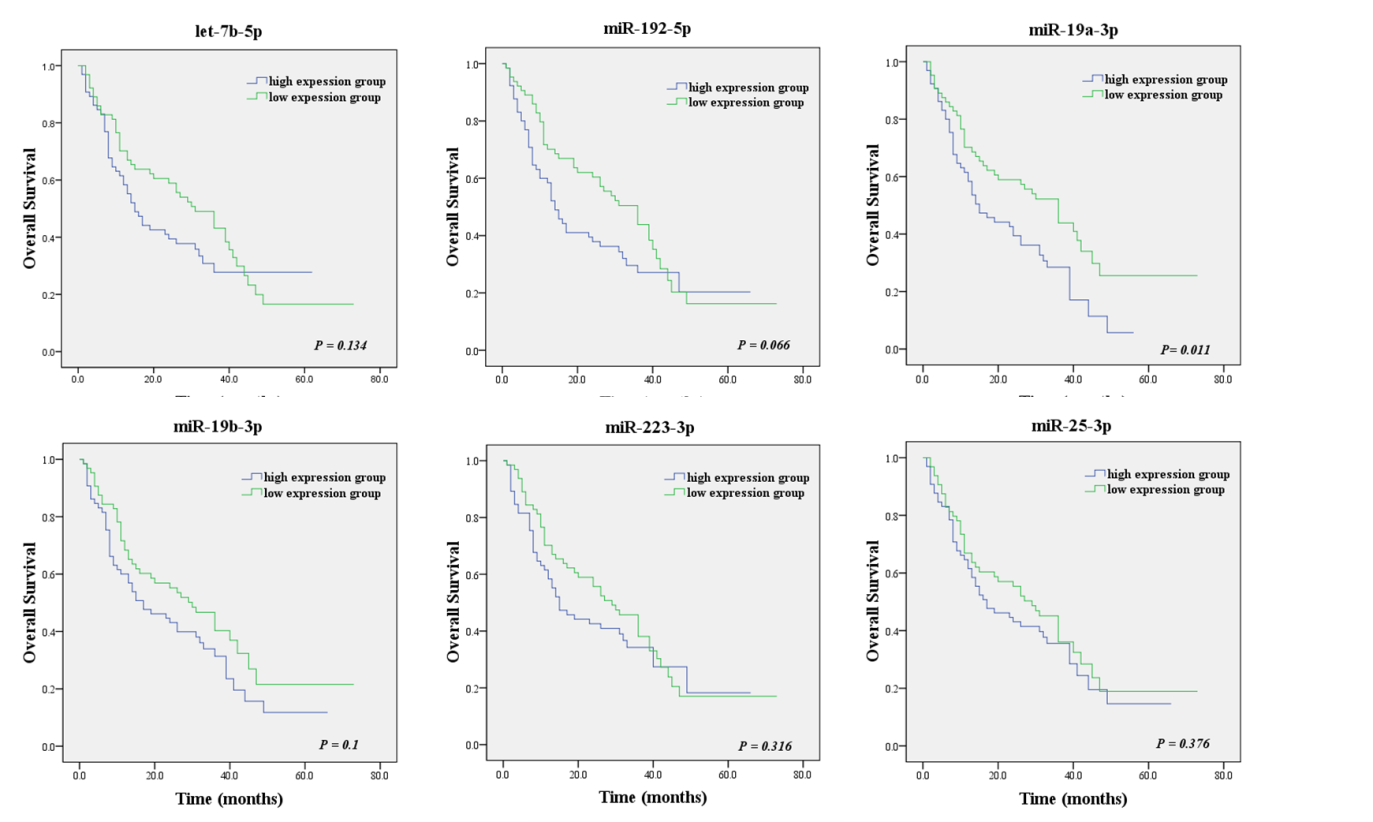

Supplement: Supplementary file 1 [file CAM4-8-2810-s001.doc]
